# Myotonometry Assessment in Children and Adolescents with Pectus Excavatum Included in a Physical Exercise Program

**DOI:** 10.3390/healthcare14050613

**Published:** 2026-02-28

**Authors:** Marius Zoltan Rezumeș, Liliana Catan, Elena Constanta Amaricai, Ada Maria Codreanu, Andreea Ancuța Vataman, Vlad Laurentiu David

**Affiliations:** 1 Doctoral School, “Victor Babeș” University of Medicine and Pharmacy, 300041 Timișoara, Romania; marius.rezumes@umft.ro (M.Z.R.); ada.codreanu@umft.ro (A.M.C.); andreea.vataman@umft.ro (A.A.V.); 2 Research Center for Assessment of Human Motion, Functionality and Disability, “Victor Babeș” University of Medicine and Pharmacy, 300041 Timișoara, Romania; amaricai.elena@umft.ro; 3 Department of Rehabilitation, Physical Medicine and Rheumatology, Faculty of Medicine, “Victor Babeș” University of Medicine and Pharmacy, 300041 Timișoara, Romania; 4 Department of Medicine, Faculty of Medicine, “Vasile Goldiș” Western University, 310038 Arad, Romania; 5 Department of Pediatric Surgery, “Victor Babeș” University of Medicine and Pharmacy, 300041 Timișoara, Romania; david.vlad@umft.ro

**Keywords:** pectus excavatum, physical therapy modalities, exercise therapy, muscle tonus, biomechanical phenomena, chest wall, posture, myotonometry, scapular stability, muscle stiffness

## Abstract

**Context/Objectives:** Pectus excavatum (PE), the most common anterior chest wall deformity in children and adolescents, impacts posture and is frequently associated with axial deviations due to biomechanical alterations of the spine and the properties of the involved musculature. **Methods:** We assessed 35 patients with PE with a Haller index below 3.25, aged between 5 and 17 years, who completed a three months specialized physical exercise program after proper training and instruction by a specialist. All patients were assessed before starting the exercise program and at the end of the treatment. The assessment method used was myotonometry, employing the MyotonPRO device, targeting the trapezius muscle with all three fascicles and the pectoralis major muscle both on the left and the right side, measuring: frequency (Hz), stiffness (N/m), decrement, relaxation time (ms), and the ratio between relaxation time and deformation time (creep). **Results:** The analysis of myotonometric parameters reveals a pattern of selective adaptation, predominantly involving the left hemibody in most of the groups analyzed, without significant functional imbalances. This asymmetry may reflect either the functional predominance of the left hemibody during participants’ daily activities or increased activation induced by the exercise program; however, by the end of the intervention, bilateral stability was observed in most parameters. **Conclusions:** A three-month physical exercise program in children and adolescents with PE results in improvements in muscle properties, particularly in the pectoralis major and middle trapezius muscles bilaterally, and contributes to the restoration of functional symmetry, thereby supporting the effectiveness of the exercise program in optimizing neuromuscular control, tissue elasticity, and scapular stability.

## 1. Introduction

Musculoskeletal disorders in children are frequently encountered by orthopedic surgeons during clinical examination, with thoracic wall malformations commonly observed [1].

Pectus excavatum (PE) is among the most common thoracic disorders, sometimes congenital, with an incidence of 23 per 10,000 births [2]. Depending on the severity, PE can cause various symptoms in the affected patient, either minor, with mild forms resulting only in esthetic concerns, while moderate forms lead to cardiopulmonary dysfunctions [3,4]. The inward deformity of the sternum can cause cardiac compression or restrictive pulmonary deficits [2,5].

The pathology in question, PE, is frequently associated with multiple axial and thoracic deviations, including an asymmetrically developed anterior thorax, anteriorly displaced shoulders, thoracic kyphosis, and, in some cases, even scoliosis, which is secondary in nature [6]. These aspects are important because such deviations alter the biomechanics of the entire body [7], with deformation of the thoracic cage itself exerting a direct impact on the spine [8]. According to this concept, alterations in the anatomical position and biomechanics of the thoracic cage, as seen in thoracic wall deformities, can lead to disturbances in spinal alignment and biomechanics [9,10].

As previously stated, although the thoracic wall plays a significant role in spinal biomechanics, spinal assessment in cases of thoracic wall deformities is often neglected, with secondary axial deviations remaining untreated [11].

The management of patients with PE using a complementary method, such as an exercise program, is undertaken following assessment and the development of a personalized plan that addresses their specific needs, including the underlying pathology and associated axial deviations; this plan is formulated by the multidisciplinary team within the Rehabilitation Department [12].

Exercise program, as the primary treatment method, employs therapeutic physical exercise whereby the musculature exerts a direct effect on the thoracic cage and spine [13], correcting muscle imbalances and consequently affecting direct correction of the bony segment to which these muscles insert [12,14].

In this context, the trapezius and pectoralis major muscles play an important role in thoracic and spinal alignment. The objective assessment of their biomechanical properties was performed using the MyotonPRO device, a non-invasive tool that allows standardized quantification of muscle tone and relevant mechanical parameters.

The objectives of the study were to evaluate changes in myotonometric parameters in children and adolescents diagnosed with pectus excavatum, before and after a three-month individualized exercise program. The assessment focused on the biomechanical and viscoelastic properties of the concave anterior thoracic musculature, as well as the trapezius and pectoralis major muscles, assessed bilaterally using the MyotonPRO device.

The study hypothesis was that the implementation of an individualized exercise program would lead to significant changes in myotonometric parameters, reflected by improvements in resting muscle tone, stiffness, elasticity, and muscle relaxation time, as well as significant differences between baseline and three-month assessments in the biomechanical and viscoelastic properties of the trapezius and pectoralis major muscles.

## 2. Materials and Methods

This study was conducted within the Medical Rehabilitation Department—Orthopedics Section of the Louis Țurcanu Clinical Emergency Hospital for Children in Timișoara, between February and October 2025. The Ethics Committee of the University of Medicine and Pharmacy of Timișoara approved the study protocol (No. 12/10.02.2025), and the study was conducted in accordance with the Declaration of Helsinki. Written informed consent was obtained from the parents after clear verbal explanations regarding each stage of the study, and participants received an informational leaflet as well as the right to withdraw at any time during the study.

### 2.1. Participants

A total of 45 patients with PE were included in this study; their ages ranged from 5 to 17 years, with the majority being male. The inclusion criteria for the study were as follows: the subject diagnosed with PE had to be a child or adolescent under 18 years of age, presenting a clinically visible minimal/mild anterior thorax concavity, radiological confirmation of the Haller index (less than 3.25), willingness to undergo the necessary evaluations, and adherence to the exercise protocol for 3 months. The initial exclusion criteria, applied to select the final 35 subjects, were as follows: age over 18 years [3], less than 3 months since any surgical intervention [1], and unwillingness to comply with the daily exercise plan [2]. Regarding the associated pathology, candidates for the study presenting neurological disorders, epilepsy [1], moderate/severe scoliosis [2], or genetic syndromes [1] were excluded.

Ten participants failed to comply with the proposed protocol; therefore, after the first selection, 35 participants remained, who completed all phases of the study according to the established methodology, and the results obtained were analyzed and included in the final conclusions.

Subjects were documented based on general characteristics, including initials, age, sex, height, weight, BMI (body mass index), as well as the Haller index, an imaging parameter quantifying the severity of thorax deformity by the ratio of the internal transverse diameter to the minimal anteroposterior distance of the thorax; the study exclusively included patients with Haller index values below 3.25, corresponding to mild and moderate forms of pectus excavatum. Additionally, thoracic circumference during maximum inspiration and expiration, as well as respiratory frequency, were assessed at the beginning and end of the exercise program.

#### Sample Size Calculation

The effect size was set at 0.5, the significance level at α = 0.05, and the statistical power at 0.8. Based on these parameters, the minimum required sample size was 34 patients, calculated using G*Power 3.1.9.7 software (Universität Kiel, Kiel, Germany), utilizing the difference between two dependent means (matched pairs). Intra-group analyses assessed changes over time, with effect size interpretation following Cohen’s conventions for large effects [15]. The sample size is comparable to that reported by Zielinski (36 patients) for studies with similar statistical parameters [16].

### 2.2. Exercise Program

Regarding the rehabilitation treatment applied to the participants, the study protocol included three months of an exercise program conducted between the initial assessment (day 0) and the final one. Immediately thereafter, on day 1, the patients began an exercise program, carried out individually by each patient, consisting of 10 exercises.

The first 10 days involved learning the correct execution of the program alongside a specialist for 60 min per day, after which the program continued at home, with each exercise performed daily in 3 sets of 10 repetitions, supervised by the parents through a journal.

The objectives of the rehabilitation program included correction and enhancement of spinal mobility, posture correction, muscular balance restoration, correction of axial deviations, and respiratory retraining.

**Figure 1:**
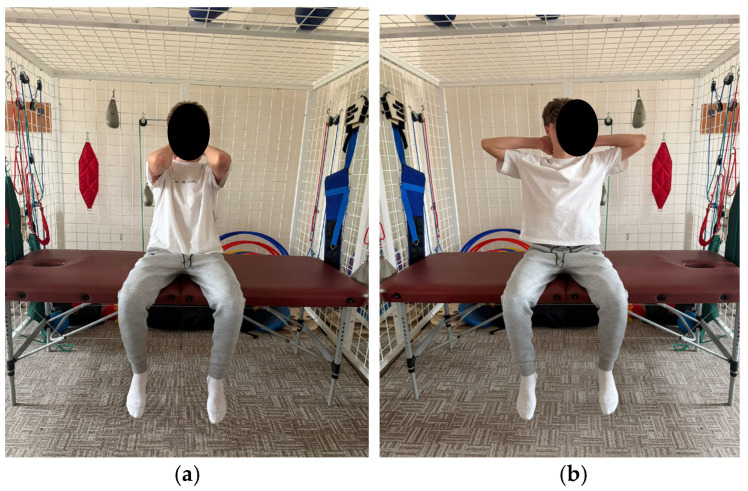
Exemplification of exercise: (**a**) initial position (**b**) final position.

**Figure 2:**
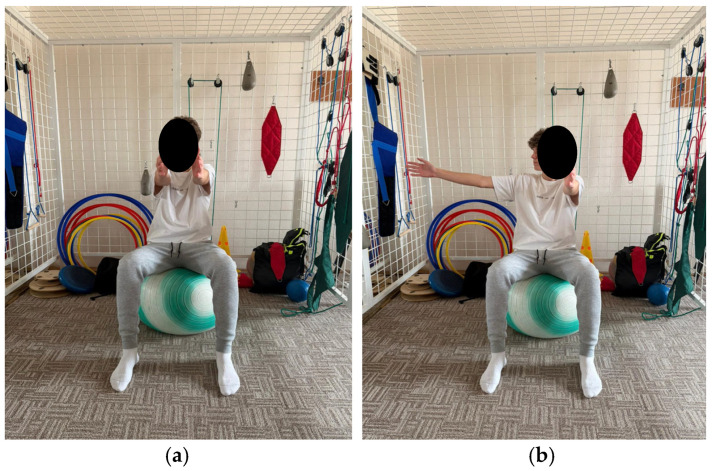
Exemplification of exercise: (**a**) initial position (**b**) final position.

**Figure 3:**
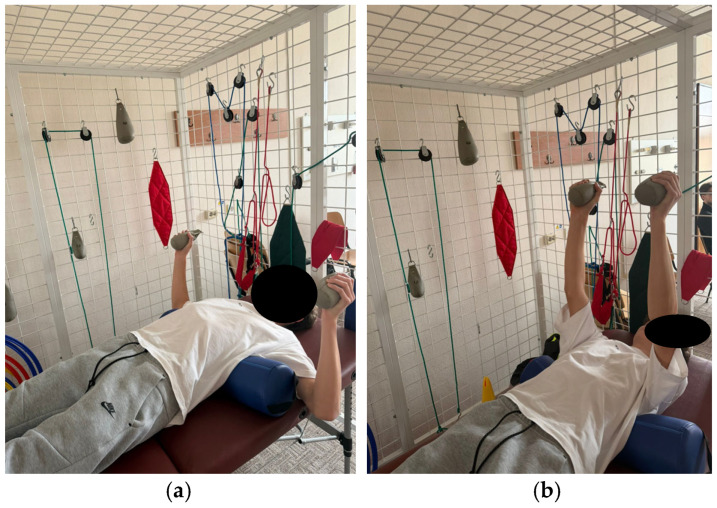
Exemplification of exercise: (**a**) initial position (**b**) final position.

**Figure 4:**
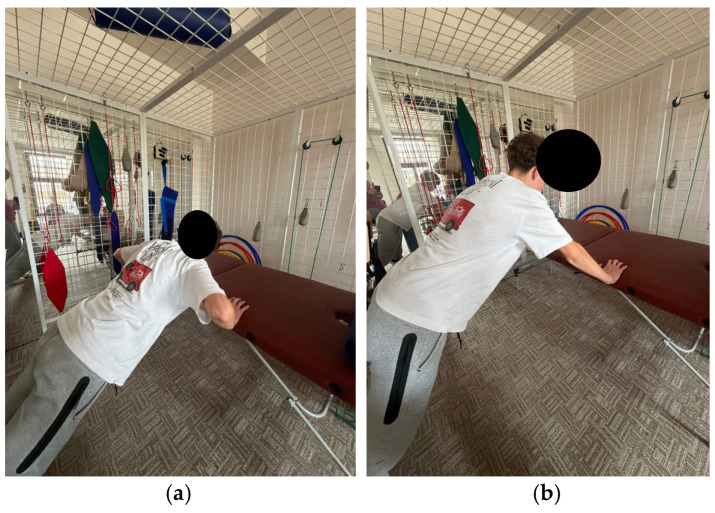
Exemplification of exercise: (**a**) initial position (**b**) final position.

**Figure 5:**
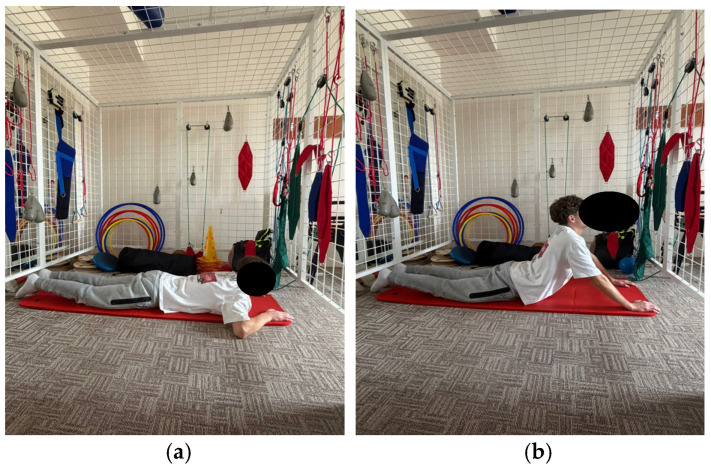
Exemplification of exercise: (**a**) initial position (**b**) final position.

The program comprises exercises aimed at improving spinal and thoracic cage mobility, as well as strengthening the trunk extensor muscles. These exercises aim to tone the back musculature through concentric training and to relax or stretch the anterior trunk muscles. Additionally, the program includes breathing retraining exercises and exercises aimed at improving stability (Figure 1, Figure 2, Figure 3, Figure 4 and Figure 5).

### 2.3. Assessment

Muscle properties were objectively assessed using the MyotonPRO device (Myoton AS, Estonia), a portable, validated, and non-invasive instrument for measuring muscle mechanical characteristics [17].

All measurements were conducted using the MyotonPRO device (Myoton AS, Tallinn, Estonia) following the manufacturer’s instructions. The device undergoes factory internal calibration and does not require additional recalibration, with only the functional integrity verified prior to each assessment session [17].

Standard mechanical impulse settings were employed (impact force of 0.4 N, duration of 15 ms, with a tissue preload of 0.18 N), and the probe was positioned perpendicular to the skin surface (tolerance ±5°) at the predetermined measurement points. For each point, a series of at least five impulses was recorded, and the measurement was considered valid if the coefficient of variation (CV) was ≤3%; otherwise, the procedure was repeated, and the mean of the accepted values was used for statistical analysis. All assessments were performed by a single trained assessor to reduce inter-assessor variability and enhance data consistency [18].

The following parameters were analyzed: frequency (Hz), stiffness (N/m), decrement, relaxation time (ms), and the ratio between relaxation time and deformation time (creep) [19].

Frequency (Hz): Represents the intrinsic muscle tone at rest. Higher values indicate a more tense or contracted muscle, whereas lower values suggest relaxation.

Stiffness (N/m): Indicates the muscle’s resistance to deformation when subjected to an external force. The higher the value, the stiffer and less elastic the muscle.

Decrement: Reflects the muscle’s ability to return to its original shape after deformation. Low values indicate good elasticity, whereas high values suggest a decrease in elasticity.

Relaxation time (ms): Represents the time required for the muscle to return to a resting state after deformation. A longer time indicates slower muscle relaxation.

Creep (the ratio between relaxation time and deformation time): Measures the muscle’s capacity to progressively adapt to sustained tension. High values indicate a more pronounced deformation over time under continuous stress [19].

For testing, each subject was seated on the electrical treatment table, which was adjusted in height so that the lower limbs were in a normal position, with the soles of the feet on the floor and the upper limbs relaxed alongside the body.

**Figure 6:**
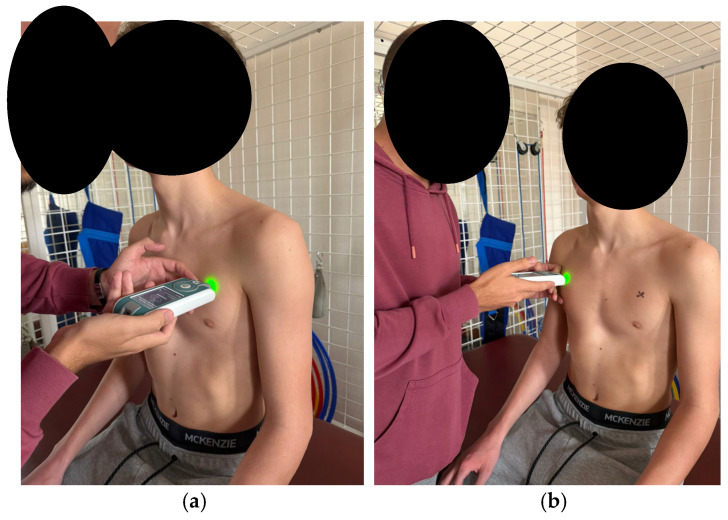
Myotonometric measurement of the pectoralis muscle (**a**) left (**b**) right.

**Figure 7:**
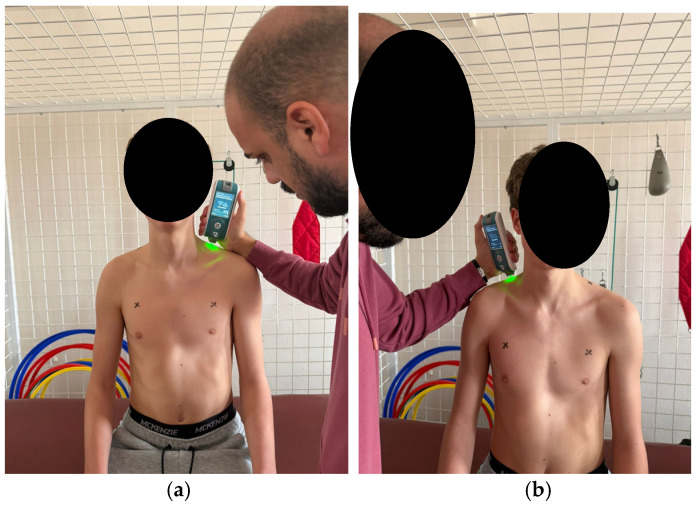
Myotonometric measurement of the upper trapezius muscle (**a**) left (**b**) right.

**Figure 8:**
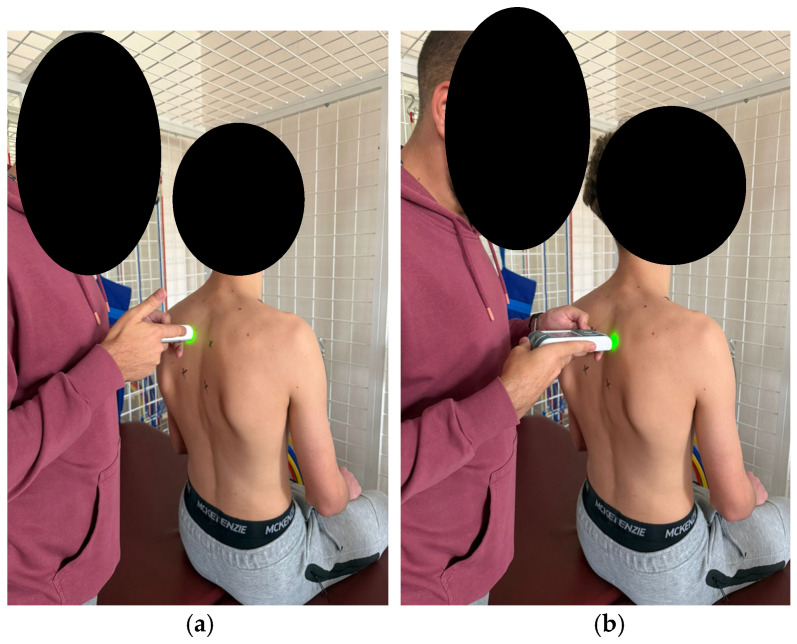
Myotonometric measurement of the middle trapezius muscle (**a**) left (**b**) right.

**Figure 9:**
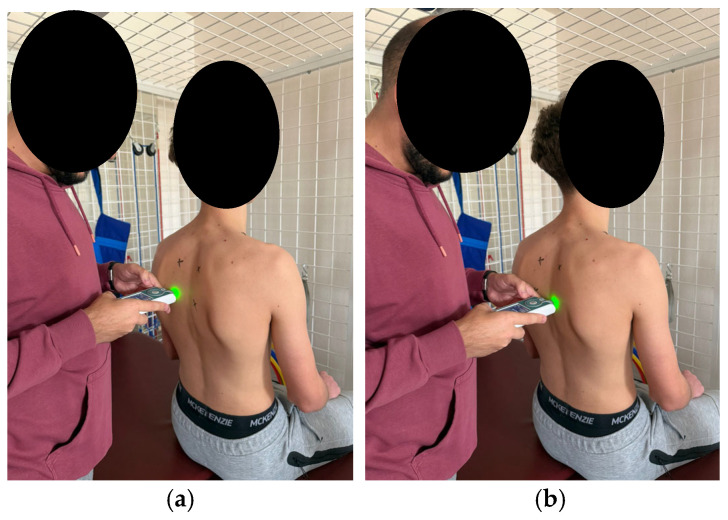
Myotonometric measurement of the lower trapezius muscle (**a**) left (**b**) right.

According to the established protocol, testing was performed bilaterally, always beginning with the left side, in the following order: pectoralis major (measured 5 cm above the nipple Figure 6), upper trapezius (perpendicular to the muscle fibers, at the midpoint between the lateral cervical line and the acromion Figure 7), middle trapezius (midpoint between the vertebral column and the medial margin of the scapula at the T3 level Figure 8), and lower trapezius (3 cm medial and inferior to the inferior angle of the scapula, along the line connecting the scapula and the spine Figure 9).

For each muscle and parameter, five consecutive measurements were obtained, and the arithmetic mean was used for data analysis.

### 2.4. Statistical Analysis

Statistical analysis was conducted using MedCalc Statistical Software, version 23.2.1 (MedCalc Software Ltd., Ostend, Belgium). For each participant, baseline (pre-intervention) and final (post-intervention) values were collected for all four muscles analyzed.

The normality of data distribution was assessed using the Shapiro–Wilk test. As the data were normally distributed, the paired-sample t-test was employed to compare baseline and final values for each muscle parameter.

For the pre–post analysis (initial vs. final), the paired samples t-test was employed, as measurements were obtained from the same subjects at two distinct time points. Thus, the *p*-value represents the probability that the observed difference between mean values is attributable to random variation rather than the intervention.

For the bilateral comparison (left vs. right), the independent samples t-test was employed, since the analysis examined differences between two distinct anatomical segments of the same individual, treated as separate variables in the statistical processing. In this context, the *p*-value indicates the presence or absence of a significant difference between the two sides.

The *p*-values reported in this study were obtained by applying appropriate inferential statistical tests for repeated measures on the same sample.

Data are presented as mean ± standard deviation (SD). The integration of the *p*-value into interpretation was carried out by direct reporting in tables and by referencing the significance threshold of *p* < 0.05. *p*-values < 0.05 were interpreted as indicating statistically significant differences, suggesting a genuine intervention-induced change or a clinically relevant bilateral imbalance, while *p*-values > 0.05 were considered not statistically significant, indicating biomechanical stability or functional symmetry.

## 3. Results

A total of 35 participants were included in the analysis, of whom 28 (80.0%) were male and 7 (20.0%) were female. The anthropometric characteristics of the group are presented in Table 1. The cohort had a mean age of 11.23 ± 3.99 years, weight of 43.14 ± 18.60 kg, height of 151.86 ± 21.70 cm, and a mean BMI of 17.77 ± 3.96 kg/m^2^, indicating a homogeneous group in terms of age, sex, and body composition.

For each participant, baseline (pre-exercise program) and final (post-exercise program) values of muscle biomechanical properties were recorded using the Myoton PRO device. Measurements were performed bilaterally (left and right) for the four muscles analyzed: upper trapezius, middle trapezius, lower trapezius, and pectoralis major. For each muscle, the five primary biomechanical parameters were assessed: Frequency (Hz), Stiffness (N/m), Decrement (log), Relaxation time (ms), and Creep (dimensionless unit).

In addition to the temporal comparison (initial vs. final) on the musculature of the same side, a bilateral analysis (left vs. right) was conducted for each muscle group to assess the presence of functional and biomechanical asymmetries. This approach is clinically relevant, as the scapular and pectoral musculature frequently present differences related to lateral dominance, postural habits, and repetitive loading patterns.

The left–right analysis enables the identification of preexisting muscular imbalances and the assessment of the intervention’s ability to restore functional symmetry. The obtained *p*-values indicate whether the adaptations induced by the intervention are distributed uniformly or preferentially unilaterally. The disappearance of significant differences at the final assessment suggests an effective process of neuromuscular rebalancing and optimization of postural control. For this comparison, only the *p*-value was reported, as the primary objective was to test the presence of statistical differences between the two sides, not to quantify the effect size.

### 3.1. Pectoralis Major (PM)

For the left pectoralis major, the pre–post analysis demonstrated significant increases in frequency (*p* = 0.0484) and stiffness (*p* = 0.0004), indicating increased muscle activation. The other parameters (decrement, relaxation, creep) did not exhibit statistically significant changes. On the right side, no significant differences were recorded between assessments for all five myotomometric parameters (Table 2).

At the initial assessment, significant differences were identified between the left and right sides, with the left side showing greater values (*p* < 0.05), indicating the presence of a clear muscular imbalance. At the final assessment, these differences were no longer significant, indicating bilateral symmetry (Table 3).

### 3.2. Upper Trapezius (UT)

In the case of the upper trapezius, the pre- and post- exercise program analysis revealed a significant increase in stiffness on the left side (*p* = 0.0138), suggesting an enhanced muscle activation. On the right side, the only significant change was an increase in decrement (*p* = 0.048), indicating a subtle alteration in viscoelastic properties. Frequency, relaxation time, and elasticity remained stable bilaterally, confirming the absence of major variations in muscle tone or flexibility (Table 4).

No significant differences were found between the left and right sides at either the initial or final assessment (*p* > 0.05), indicating maintenance of symmetry (Table 5).

### 3.3. Middle Trapezius (MT)

The left middle trapezius demonstrated significant changes characterized by decreased stiffness (*p* = 0.0472) and significant increases in relaxation time and elasticity (*p* < 0.001). These changes indicate reduced muscle tension and improved elastic properties. Meanwhile, the right side shows stable parameters, without statistically significant changes, contributing to symmetric thoracic alignment (Table 6).

Initially, no significant differences were observed between the right and left sides. However, at the final evaluation, significant differences appeared in the parameters on the right side (*p* < 0.05), suggesting a more pronounced unilateral adaptation, possibly influenced by lateral dominance or individual functional patterns (Table 7).

### 3.4. Lower Trapezius (LT)

For the lower trapezius, the only significant change was an increase in decrement on the left side (*p* = 0.028), indicating a modification in the damping of muscular vibrations. All other parameters, including frequency, stiffness, relaxation time, and elasticity, remained stable on both sides, suggesting the absence of major mechanical adjustments in response to the intervention (Table 8).

During the initial assessment, significant differences between the sides were identified, with the left side showing statistically significant values compared to the right for decrement and relaxation, indicating a preexisting muscular imbalance. At the final assessment, these differences were no longer significant, suggesting progressive neuromuscular rebalancing (Table 9).

## 4. Discussion

The primary objective of this study was to assess the biomechanical changes in the musculature involved in scapular and shoulder stability following a three-month exercise program in children with PE.

No studies evaluating the properties of muscles involved in the development of PE were identified in our searches; however, associations between this type of sternal deformity and thoracic kyphosis have been observed [20], with limitations in thoracic spinal mobility potentially caused by alterations in both contractile and non-contractile tissues [21].

Qiu-Shuo Tian et al., in a study published in 2025, presented the results obtained through myotonometric assessment of the sternocleidomastoid and upper trapezius muscles, utilizing MyotonPRO in 32 subjects with cervical extension, characterized by increased cervical lordosis, forward head posture, and thoracic kyphosis, who completed a four-week exercise program.

They observed significant differences in muscle tone, stiffness, and elasticity, demonstrating that the exercises performed had a positive effect not only on myotonometric parameters but also on posture, pain, and cervical stability [22].

An abnormal, elevated position in the upper thoracic region of the pectoralis major muscle, which biomechanically elevates the sternum, constitutes a potential causal factor for PE [23].

Due to the absence of studies investigating the properties of muscles involved in the development and progression of PE, the following analysis of the present study’s results is provided, based on four muscles assessed before and after the three months of therapeutic intervention through an exercise program.

At the initial assessment of the pectoralis major muscle, significant bilateral asymmetries were detected across multiple parameters, indicating a pronounced muscular imbalance between the left and right sides. These differences suggest distinct biomechanical behavior of the two hemibodies, likely associated with postural characteristics or functional dominance.

However, at the final assessment, the differences between the two sides were no longer statistically significant, indicating a functional rebalancing of the pectoral musculature and restoration of muscular symmetry as a result of the therapeutic intervention.

The increase in frequency and stiffness of the left pectoralis major muscle suggests improved muscle tone and contractile capacity, possibly as a response to the exercises or a more efficient repositioning of the scapulohumeral joint. The stability of parameters on the right side indicates the maintenance of muscular balance without overload. These adaptations are favorable for pushing function and anterior shoulder stability in children with PE who were assessed.

The upper trapezius muscle demonstrates increased stiffness on the left side, which may reflect more efficient muscle activation and improved superior scapular stability, both beneficial for cervical postural control. The slight alteration of viscoelastic parameters on the right side suggests subtle tissue adaptation without significant functional implications. The stability of the remaining parameters indicates the maintenance of bilateral muscular balance, which is favorable for preventing compensations and overload at the shoulder and neck levels.

For the upper trapezius muscle assessed in this study, no significant differences were observed between the left and right sides for any parameter, either initially or at the end. This suggests good muscular symmetry and functional stability of the upper trapezius throughout the study.

Compared to the evaluated upper trapezius muscle, the results suggest normalization of muscle tone in the left middle trapezius, which is beneficial for scapular mobility and reduction in tension in the upper thoracic region. The increase in elasticity and relaxation time reflects an improvement in muscle flexibility and recovery capacity. The right side maintains stable values, indicating functional equilibrium without unilateral overload.

Although no significant asymmetries were initially observed between the two sides of the upper trapezius muscle, the final assessment reveals significant differences in frequency, stiffness, and creep, suggesting the development of unilateral functional predominance (likely on the side more engaged in daily activities). This may represent an adaptive pattern specific to the intervention or a result of preferential use.

At the level of the lower trapezius muscle, an increase in the left decrement was observed, which may reflect an improvement in the neuromuscular control of this muscle, essential for scapular stabilization during upward rotation and for maintaining healthy shoulder posture. The stability of the other parameters indicates a balanced biomechanical profile without signs of overuse or compensatory mechanisms.

Although initially the lower trapezius muscle exhibited significant asymmetries between the left and right sides in terms of decrement and relaxation, indicating a preexisting neuromuscular imbalance, at the final assessment, these differences were no longer significant, suggesting normalization of neuromuscular control and improvement of bilateral balance.

The results obtained in this study through the myotonometric assessment of children with PE indicate a selective adaptation pattern, predominantly involving the left hemibody in most groups analyzed, but without significant functional imbalances. This asymmetry may reflect either the functional dominance of the left hemibody in the participants’ daily activities or an increased activation induced by the exercise program. However, the bilateral stability observed at the conclusion of the intervention across most parameters suggests that the three months of exercise program were well tolerated and contributed to improvements in neuromuscular control, tissue elasticity, and scapulohumeral function.

The exercise program applied to the 35 children with PE participating in the study was the one described in the methods section and included elements similar to those implemented in other studies, such as the one published by Davi DE Podestá Haje et al., in which participants performed specific strengthening exercises for the anterior thoracic muscles at least five times per week, reporting results that demonstrated the efficacy of these exercises for the correction or partial correction of PE, particularly when therapeutic intervention was initiated early in milder and more flexible deformities [24]. The combination of cardiopulmonary exercises, encompassing upper lateral costal and middle/lower diaphragmatic breathing, as well as respiratory exercises targeting each pulmonary lobe, in addition to musculoskeletal system exercises for stretching, strengthening, postural awareness, trunk mobilization, and manipulation, along with supplementary aerobic exercises, recommended by Nuray Alaca et al. in their 2020 study for patients with PE [25], was also incorporated in the present study.

In our study, we recorded a higher frequency of the pectoralis major after the 3-month exercise program. The frequency characterizes the muscle tone in the resting state. There were no significant differences between the right and left tone of the pectoralis major after the exercise program. The increased tone of an accessory respiratory muscle, with no significant difference between the right and left sides, suggests an improved respiratory capacity after the 3-month exercise program. The decrease in stiffness of the middle trapezius after completing the exercise program supports improved scapular positioning and a more balanced posture compared to the initial assessment. Meanwhile, the decrease in the pectoral, an accessory respiratory muscle, may reflect reduced muscle elasticity and adaptations that can enhance respiratory mechanics at the end of the program. These changes suggest that the exercise program provides functional and postural benefits in addition to local effects on the muscles.

### Limitations

The relatively small sample size, single-center study design, and differences in gender and age represent the limitations of the current study. Although a similar therapeutic approach was applied in all cases, variations in patient cooperation and compliance may have influenced the results. Another limitation is the lack of standardization of the exercise program, as each patient followed an individualized protocol based on age and permitted complexity, which may have introduced variability in the recovery process. Despite these limitations, a focused set of inclusion and exclusion criteria allowed a more accurate analysis of myotonometric parameters.

The main limitation of this study is the absence of a control group, which restricts the ability to attribute the observed changes exclusively to the intervention. However, the objective was to evaluate the effects of the exercise program in the same participants by comparing pre- and post- exercise program results. Future studies including a randomized control group are needed to confirm causal relationships.

## 5. Conclusions

The implementation of a 3-month exercise program in children and adolescents with PE leads to improvements in muscle properties, especially in the pectoralis major and middle trapezius muscles bilaterally, and plays a role in restoring functional symmetry, thus supporting the effectiveness of the exercise program in optimizing neuromuscular control, tissue elasticity, and scapular stability.

## Figures and Tables

**Table 1:** The anthropometric characteristics of the group are presented.

Variable	Mean ± SD
age (years)	11.23 ± 3.99
weight (kg)	43.14 ± 18.60
height (cm)	151.86 ± 21.70
BMI (kg/m^2^)	17.77 ± 3.96

cm: centimeter; kg: kilogram; BMI: body mass index; SD: standard deviation.

**Table 2:** Myotonometric assessment of the pectoralis major pre- and post-exercise program (3 months).

Parameter	Left Side			Right Side		
	Initial	Final	*p*-Value	Initial	Final	*p*-Value
Frequency (Hz) (mean ± SD)	11.48 ± 1.71	12.26 ± 2.56	0.0484 *	12.35 ± 2.28	12.34 ± 2.49	0.9609
Stiffness (N/m) (mean ± SD)	204.00 ± 77.05	243.51 ± 95.68	0.0004 *	233.63 ± 84.53	245.66 ± 87.47	0.2012
Decrement (mean ± SD)	1.38 ± 0.43	1.39 ± 0.53	0.3932	1.45 ± 0.60	1.40 ± 0.46	0.1736
Relaxation (ms) (mean ± SD)	22.45 ± 6.33	20.49 ± 5.20	0.0810	24.54 ± 9.07	23.27 ± 7.47	0.1736
Creep (mean ± SD)	1.23 ± 0.27	1.13 ± 0.40	0.0954	1.40 ± 0.47	1.39 ± 0.48	0.7388

SD: standard deviation; Hz: hertz; N/m: newton per meter; ms: millisecond; *p*-value: probability value; *: statistically significant result.

**Table 3:** Myotonometric assessment of the Pectoralis Major—Left vs. Right.

Parameter	*p*-Value Initial	*p*-Value Final
Frequency (Hz)	0.0008 *	0.0558
Stiffness (N/m)	0.0193 *	0.3354
Decrement	0.4306	0.9078
Relaxation (ms)	0.0095 *	0.2040
Creep	0.0154 *	0.1931

Hz: Hertz; N/m: newton per meter; ms: millisecond; *p*-value: probability value; *: statistically significant result.

**Table 4:** Myotonometric assessment of the upper trapezius pre- and post- exercise program (3 months).

Parameter	Left Side			Right Side		
	Initial	Final	*p*-Value	Initial	Final	*p*-Value
Frequency (Hz) (mean ± SD)	15.80 ± 1.93	15.88 ± 1.86	0.7490	12.83 ± 2.39	12.85 ± 2.04	0.9600
Stiffness (N/m) (mean ± SD)	308.40 ± 34.72	324.77 ± 68.20	0.0138 *	302.17 ± 55.10	319.46 ± 68.50	0.2972
Decrement (mean ± SD)	0.98 ± 0.18	1.02 ± 0.18	0.0863	1.03 ± 0.22	1.10 ± 0.21	0.0480 *
Relaxation (ms) (mean ± SD)	16.63 ± 2.13	16.28 ± 2.16	0.2135	15.69 ± 1.93	15.80 ± 2.01	0.3363
Creep (mean ± SD)	0.99 ± 0.11	0.97 ± 0.19	0.5720	0.99 ± 0.15	1.03 ± 0.20	0.4164

SD: standard deviation; Hz: hertz; N/m: newton per meter; ms: millisecond; *p*-value: probability value; *: statistically significant result.

**Table 5:** Myotonometric assessment of the upper trapezius—Left vs. Right.

Parameter	*p*-Value Initial	*p*-Value Final
Frequency (Hz)	0.5671	0.4621
Stiffness (N/m)	0.1799	0.6048
Decrement	0.4035	0.8356
Relaxation (ms)	0.1739	0.7028
Creep	0.0750	0.3909

Hz: Hertz; N/m: newton per meter; ms: millisecond; *p*-value: probability value.

**Table 6:** Myotonometric assessment of the Middle Trapezius pre- and post- exercise program (3 months).

Parameter	Left Side			Right Side		
	Initial	Final	*p*-Value	Initial	Final	*p*-Value
Frequency (Hz) (mean ± SD)	17.32 ± 2.54	16.79 ± 1.95	0.1411	15.83 ± 2.05	15.71 ± 1.84	0.7376
Stiffness (N/m) (mean ± SD)	398.57 ± 106.28	367.94 ± 80.87	0.0472 *	381.14 ± 91.96	354.37 ± 75.88	0.0786
Decrement (mean ± SD)	1.04 ± 0.26	1.14 ± 0.23	0.1224	1.10 ± 0.35	1.21 ± 0.35	0.0736
Relaxation (ms) (mean ± SD)	13.68 ± 2.56	14.69 ± 2.46	0.0005 *	14.77 ± 3.28	14.00 ± 2.49	0.1451
Creep (mean ± SD)	0.83 ± 0.16	0.92 ± 0.19	0.0004 *	0.91 ± 0.25	0.90 ± 0.25	0.8985

SD: standard deviation; Hz: hertz; N/m: newton per meter; ms: millisecond; *p*-value: probability value; * statistically significant result.

**Table 7:** Myotonometric assessment of the middle trapezius—Left vs. Right.

Parameter	*p*-Value Initial	*p*-Value Final
Frequency (Hz)	0.4690	0.0105 *
Stiffness (N/m)	0.6587	0.0392 *
Decrement	0.2632	0.0582
Relaxation (ms)	0.6495	0.2199
Creep	0.8703	0.0099 *

Hz: Hertz; N/m: newton per meter; ms: millisecond; *p*-value: probability value; *: statistically significant result.

**Table 8:** Myotonometric assessment of the lower trapezius pre- and post- exercise program (3 months).

Parameter	Left Side			Right Side		
	Initial	Final	*p*-Value	Initial	Final	*p*-Value
Frequency (Hz) (mean ± SD)	13.57 ± 2.11	13.64 ± 1.91	0.4529	13.40 ± 2.16	13.19 ± 1.96	0.5012
Stiffness (N/m) (mean ± SD)	412.83 ± 126.61	404.91 ± 102.26	0.6477	415.69 ± 122.30	394.00 ± 95.06	0.3424
Decrement (mean ± SD)	1.09 ± 0.16	1.15 ± 0.21	0.0280 *	1.14 ± 0.24	1.15 ± 0.24	0.7437
Relaxation (ms) (mean ± SD)	14.43 ± 2.49	14.57 ± 1.99	0.7388	14.64 ± 2.45	14.64 ± 2.40	0.9648
Creep (mean ± SD)	0.88 ± 0.22	0.89 ± 0.20	0.6074	0.87 ± 0.17	0.88 ± 0.17	0.7330

SD: standard deviation; Hz: hertz; N/m: newton per meter; ms: millisecond; *p*-value: probability value; *: statistically significant result.

**Table 9:** Myotonometric assessment of the lower trapezius—Left vs. Right.

Parameter	*p*-Value Initial	*p*-Value Final
Frequency (Hz)	0.7795	0.0670
Stiffness (N/m)	0.8767	0.0576
Decrement	0.0053 *	0.0994
Relaxation (ms)	0.0190 *	0.4480
Creep	0.1797	0.0536

Hz: Hertz; N/m: newton per meter; ms: millisecond; *p*-value: probability value; *: statistically significant result.

## Data Availability

The data presented in this study are available on request from the corresponding author (L.C.) due to privacy.

## Abbreviations

The following abbreviations are used in this manuscript:

PE	Pectus excavatum
Hz	Hertz
N/m	Newton per meter
ms	Millisecond
BMI	Body mass index
SD	Standard deviation
CV	Coefficient of variation
UT	Upper trapezius
MT	Middle trapezius
LT	Lower trapezius
PM	Pectoralis major
Fig.	Figure
No.	Number
*p*-value	Probability value
G power	Statistical power analysis software
T3	Thoracic vertebra 3
AS	Company designation
Kg	Kilogram
Cm	Centimeter
